# Failure to confirm allelic and haplotypic association between markers at the chromosome 6p22.3 dystrobrevin-binding protein 1 (DTNBP1) locus and schizophrenia

**DOI:** 10.1186/1744-9081-3-50

**Published:** 2007-09-23

**Authors:** Susmita R Datta, Andrew McQuillin, Vinay Puri, Khalid Choudhury, Srinivasa Thirumalai, Jacob Lawrence, Jonathan Pimm, Nicholas Bass, Graham Lamb, Helen Moorey, Jenny Morgan, Bhaskar Punukollu, Gomathinayagam Kandasami, Simon Kirwin, Akeem Sule, Digby Quested, David Curtis, Hugh MD Gurling

**Affiliations:** 1 Molecular Psychiatry Laboratory, Department of Mental Health Sciences, University College London Medical School, Windeyer Institute of Medical Sciences, 46 Cleveland Street, London, W1T 4JF, UK; 2 West Berkshire NHS Trust, 25 Erleigh Road, Reading, RG3 5LR, UK; 3 Camden and Islington Mental Health and Social Care Trust, St Pancras Hospital, London, NW1 0PE, UK; 4 West London Mental Health Trust, Hammersmith & Fulham Mental Health Unit and St Bernard's Hospital, London, W6 8RF, UK; 5 Department of Psychiatry, University of Oxford, Warneford Hospital, Headington, Oxford, UK; 6 Queen Mary College, University of London and East London and City Mental Health Trust, Royal London Hospital, Whitechapel, London, E1 1BB, UK; 7 Mersey Care NHS Trust, University Hospital Aintree, Longmoor Lane, Aintree, Liverpool, L9 7AD, UK; 8 Hampshire Partnership NHS Trust, Mulfords Hill Centre, Tadley, Hampshire, RG26 3HX, UK

## Abstract

**Background:**

Previous linkage and association studies may have implicated the Dystrobrevin-binding protein 1 (DTNBP1) gene locus or a gene in linkage disequilibrium with DTNBP1 on chromosome 6p22.3 in genetic susceptibility to schizophrenia.

**Methods:**

We used the case control design to test for of allelic and haplotypic association with schizophrenia in a sample of four hundred and fifty research subjects with schizophrenia and four hundred and fifty ancestrally matched supernormal controls. We genotyped the SNP markers previously found to be significantly associated with schizophrenia in the original study and also other markers found to be positive in subsequent studies.

**Results:**

We could find no evidence of allelic, genotypic or haplotypic association with schizophrenia in our UK sample.

**Conclusion:**

The results suggest that the DTNBP1 gene contribution to schizophrenia must be rare or absent in our sample. The discrepant allelic association results in previous studies of association between DTNBP1 and schizophrenia could be due population admixture. However, even positive studies of European populations do not show any consistent DTNBP1 alleles or haplotypes associated with schizophrenia. Further research is needed to resolve these issues. The possible confounding of linkage with association in family samples already showing linkage at 6p22.3 might be revealed by testing genes closely linked to DTNBP1 for allelic association and by restricting family based tests of association to only one case per family.

## Background

Schizophrenia has a life time prevalence of 0.85% in the United Kingdom. Family, twin and adoption studies have shown that there is a strong genetic susceptibility to schizophrenia. Multiply affected families are common, and there is good evidence that genes increase susceptibility to schizophrenia, and can be transmitted by obligate carriers with no formal mental illness to affected offspring and that the genetic heritability lies between 66% and 93% [1]. Clinically unaffected obligate carriers as well as "normal" monozygotic co-twins have repeatedly been shown to possess an "endophenotype" of brain volumetric deficit, abnormal smooth pursuit eye movements, abnormal evoked EEG responses and abnormal patterns of speech [1]. Replicated evidence from genetic linkage studies has confirmed that multiple chromosomal loci are involved in the heritability of the disease.

Evidence from linkage analysis in families that there is a susceptibility locus for schizophrenia on chromosome 6p22.3 has been provided by several groups [2-5]. However, we and several other research groups were unable to detect the presence of this locus in other family samples [6-10]. The variability in the outcome of linkage studies has been attributed to heterogeneity of linkage. This implies that individual families have genetic effects not shared by other families and that there are multiple different genetic subtypes of schizophrenia. Confirmed evidence for linkage at the 6p22.3 locus was followed up with allelic association studies using the family based methods implemented in TRANSMIT [11]. With this method it is theoretically possible to confound linkage with association unless a strict test of transmission disequilibrium is carried out by using only one affected case in a family together with one set of parents. In general the potential for confounding of linkage with association is most likely when a family sample has already shown linkage to the markers for which there is apparent evidence for association and when the commonest alleles and haplotypes appear to be showing association. In the case of the original report of allelic association between DTNBP1 gene and schizophrenia the same family sample was already known to show good evidence for linkage. An admixture test on lod scores from the linkage analysis was significant in rejecting homogeneity of linkage and was also significant for showing significant admixture in the family sample. Nevertheless when the most stringent test of allelic association was carried out by observing transmission disequilibrium of alleles into one case of schizophrenia per family per kindred in the sample of 270 Irish families evidence for allelic association with schizophrenia was weakened but still present [11]. This was computed with TRANSMIT but other family based tests of association have also been which could still have confounded linkage with association.

The haplotype structure in the original Irish families showed that 96% of the variability in the sample was attributed to six haplotypes [12]. Only one of the six haplotypes was identified as a high risk haplotype and this had a frequency of 6% in the whole sample [12]. The consistency of the disease haplotypes associated with schizophrenia are further explored in the discussion section of this paper in the light of work by Mutsuddi and coworkers [13]. These researchers compared all published haplotypes in the Centre d'Etude du Polymorphisme Humain (CEPH) reference DNA samples with those in published association studies.

A subsequent study of 78 German, Hungarian and Israeli families also showed positive tests of allelic transmission disequilibrium with the DTNBP1 locus but only with a single SNP and not with any of the other SNPs that had been previously found to be associated in the Irish family sample [14]. The German sample also showed a positive haplotypic association composed of three adjacent SNPs. However, the alleles in the associated haplotype were different to those found in Straub et al's Irish sample [11].

A positive haplotypic association was also found in a study of 233 Han Chinese trios, where the risk haplotype was composed of the most common SNP alleles [15]. This was in contrast to the haplotype associated in the Irish study which was composed of minor SNP alleles [11]. A genetic association study consisting of three case control samples from Germany, Poland and Sweden found no evidence for association with any of the five SNPs previously reported to be positive except for one SNP (rs1011313, p = 0.032) in the Swedish sub sample. Haplotypic associations were also negative for the German and Polish sub samples. Only the Swedish schizophrenic sample showed haplotypic association with schizophrenia. Several, two, three or four marker haplotypes and a single 5-marker SNP haplotype showed association. The strength of association was increased in the subgroup of cases with a positive family history of schizophrenia. The 5 marker haplotype consisted of the nucleotide alleles A-C-A-T-T at P1635; P1325; P1757; P1320 and P1578 respectively (3.1% in controls 17.7% in cases) [16]. It should be noted that the Swedish sub sample only contained 32 family history positive cases which means that the standard error for these statistical tests was high.

The haplotype associated in the original Straub et al [11] data was G-C-A-T-C and in the Swedish sub sample was A-C-X-C-C, where X is an SNP not tested in the Swedish sub sample (P1635; P1325; P1757; P1320; P1578). The Swedish haplotype is yet another one to be associated with schizophrenia. Van Den Bogaert et al., [16] suggest that the 5 marker Swedish haplotype associated with schizophrenia is phylogenetically related to the original haplotype found to be associated with Irish schizophrenia by Straub et al [11]. However, the absence of clear cut association in this sample combined with inconsistent haplotypes being associated with schizophrenia does not encourage strong belief that this study has confirmed the original data by Straub et al [11].

A novel DTNBP1 haplotype incorporating SNP A (T allele) has been identified in a case control sample of 708 cases and 711 controls of British and Irish descent that shows association with schizophrenia [17]. This haplotype was also found to be associated with schizophrenia in an Irish case control sample that did not initially show association [18,19]. This haplotype consisted of one novel SNP (SNP A, A allele trend for increase in cases p = 0.06) in combination with P1655 (p = 0.91) and P1635 (p = 0.82) from the Straub study [11] and gave an empirical p value of 0.000056. The discrepancy between the significance of the SNP A single marker association with schizophrenia and the significance of the haplotypic association is unexpected. Secondly the SNP A T allele showed a trend towards association when single markers were tabulated but the T allele was increased in the cases as part of the haplotypes associated with schizophrenia. The same research group also reported a positive replication in a sample of 488 Hungarian schizophrenic trios [19]. However, the two markers and alleles showing association were P1325 A allele and the P1757 G allele. The two alleles associated in the original study were the G and A alleles at P1325 and P1757. Once again there is an inconsistency in the identity of the alleles and haplotypes associated with schizophrenia in the different studies. It seems that the study by Kirov et al (2004) [19] found association between two loci and schizophrenia with the opposite alleles to those found to be associated in the original Irish study. This is inconsistent with there being an evolutionary relationship between a schizophrenia susceptibility mutation in different European sub populations.

A case control study by Funke and colleagues analysed 7 SNPs from the original Straub study [11] in a sample of 524 patients with schizophrenia or schizoaffective disorder and 573 control subjects [20]. This sample was split into white, Hispanic and African American sub-samples. In the white subset of 258 patients and 467 controls, the opposite allele T at P1578 to the one found associated in the original Straub et al (2002) study (allele C) was associated with schizophrenia. At P1763 the A allele was associated in the study by Funke et al [20] but it was the C allele that was originally associated by Straub et al [11]. Only the A allele at P1765 was consistently associated with schizophrenia in the original and Funke studies. The Hispanic subset (51 cases and 32 controls) was also found to have the same 3 SNPs associated with schizophrenia with the same inconsistencies with the original study. No association was observed in the African American subset which consisted of 215 cases and 74 controls.

Hall et al [21] investigated the role of DTNBP1 in schizophrenia in 210 US trios and 169 trios of Afrikaans descent. Neither population displayed any significant allelic or haplotypic associations between DTNBP1 and schizophrenia [21].

An Israeli isolate was used in a genome wide scan by Kohn and colleagues to identify loci predisposing to any diagnosis of psychosis [22]. This isolate was unique in that there was only one paternal origin for the whole village as a result of a bottleneck event that occurred a few centuries ago. IBD haplotype sharing was used to carry out a genome wide scan with 359 microsatellite markers. A positive linkage (p < 0.0001) was observed over the DTNBP1 gene on chromosome 6. However, such an inbred population cannot distinguish between linkage and association.

Numakawa and colleagues used a case control sample of 670 patients and 588 controls and found that four SNPs, P1635 (G allele) P1320 (T allele) P1763 (G allele) and SNP A (A allele) were associated with schizophrenia [23]. The same authors also detected positive haplotypic association (p = 0.00028). The original alleles associated with schizophrenia were the G, T, C and T alleles respectively. Thus once again inconsistencies in the alleles and haplotypes found to be associated are apparent. There is also the problem that the haplotypic associations are so much stronger than the individual SNP association. P values with relatively less differences between single marker and haplotypic association would be expected.

Positive allelic and haplotypic associations were also reported by Li and colleagues in a Han Chinese population [24]. Positive haplotypic but not allelic association was reported by Tochigi et al, [25]. However, as with the some other studies [14,15], the associated haplotypes were not the same as reported in the original study. Li et al [24] screened a Scottish sample of 580 schizophrenic cases and 620 controls. They were unable to replicate any previously identified single marker or haplotypic associations. In particular the finding of a protective haplotype of SNPA-P1655-P1635 was not replicated [17]. This haplotype was found to be strongly associated in a British population and was nominally significantly associated in an Irish study, therefore one might expect a similar effect in a Scottish population. However, only a partial replication in a Scottish sample, with a rare haplotype of SNP A with P1583 remained significant after permutation testing (p = 0.03) [24].

DTNBP1 is a 40-kDa coiled-coil-containing protein that shows cross species evolutionary conservation [26]. DTNBP1 binds to both alpha- and beta-dystrobrevin in muscle and brain [26]. In the brain DTNBP1 is found in axons, particularly in the mossy fiber synaptic terminals of the cerebellum and hippocampus [26]. Talbot and colleagues compared presynaptic dysbindin-1 levels at hippocampal formation sites lacking neuronal dystrobrevin between schizophrenics and controls and found that these levels were significantly reduced in schizophrenics [27].

Weickert and colleagues [28] have studied the role and distribution of dysbindin in brains from schizophrenics and controls. Dysbindin mRNA levels in the frontal cortex, temporal cortex, hippocampus, caudate, putamen, nucleus accumbens, amygdala, thalamus, and midbrain were significantly reduced in schizophrenic brains. In the midbrain there was a reduction of dysbindin in patients, but this was not statistically significant. Significant genotype dependent differences in cortical levels of dysbindin were also observed. This finding was supported by Bray and colleagues in Cardiff [29] who demonstrated that a schizophrenia risk haplotype tagged one or more cis acting variants that reduced expression of DTNBP1 in post mortem cerebral cortex mRNA of unaffected research subjects. Numakawa et al (2004) investigated the functions of dysbindin in primary neuronal cultures. They found that over expression of dysbindin induced the expression of two presynaptic proteins, SNAP25 and synapsin 1. Increased extracellular basal glutamate levels and glutamate release was also observed as a result of dysbindin over expression and this was thought to be evoked by high potassium levels. Reduced expression of dysbindin using small interfering RNA (siRNA) caused the opposite effect with a reduction of pre-synaptic proteins and glutamate. The authors suggested that the glutamate increase was a direct effect of dysbindin up regulating the pre-synaptic molecules. Another effect of over expressing dysbindin was an increase in the phosphorylation of AKT1, a gene that itself has been associated with schizophrenia [30,31].

A possible role for DTNBP1 in both bipolar disorder and major depression was investigated in three studies, single marker allelic association with the SNPs identified by Straub et al[11] was not detected in any of these studies [32-34]. However, Breen et al., [34] reported genotypic association with P1757 and P1320 and they also found haplotypic association with markers P1765, P1757, P1320 and P1763. In addition, DTNBP1 has also been implicated in Duchenne muscular dystrophy [26] and in Hermansky-Pudlak syndrome type 7 (HPS-7) [35].

## Methods

### Clinical sampling

450 cases of schizophrenia were studied together with 450 screened normal controls. The cases and controls were all asked if both their parents and all four grandparents were of Irish, Welsh, Scottish or English ancestry using our ancestry questionnaire. Those subjects with two or more grandparents who had any other ancestry were excluded but subjects with a single grandparent with European Union (EU) on the basis of the EU countries before 2004 enlargement), but non Jewish ancestry were included. U.K. National Health Service (NHS) multicentre and local research ethics committee approval was obtained for this study. All subjects gave written consent after reading an approved information sheet. All affected and unaffected subjects were interviewed by a psychiatrist using the Lifetime Version of the Schizophrenia and Affective Disorders Schedule (SADS-L) [36]. The cases were also rated using the 90-item OPCRIT checklist [37], the family history was recorded and a pedigree diagram was drawn. Diagnoses were assigned using Research Diagnostic Criteria [38]. 60 ml of blood sample was taken from each individual for DNA analysis. Genomic DNA was extracted using the phenol-chloroform method.

### Genotyping

All SNPs were genotyped by KBiosciences (Hertfordshire, UK) which employs either the Amplifluor, KASPar or Taqman SNP genotyping methods or were genotyped by us using Pyrosequencing. Internal controls were used to check for accuracy with 17% of samples duplicated in order to detect error and confirm the reproducibility of genotypes. The reduplication of sample genotyping was carried out in a separate microtitre plate and was PCR amplified separately.

### Sequencing

The region incorporating SNPs P1757 and P1320 was resequenced by us in the molecular psychiatry laboratory at UCL in random individuals to validate the results of genotyping from Kbioscience. Direct genomic PCR based sequencing was carried out using M13 tailed primers synthesised to be homologous to genomic DNA sequences flanking either side of the polymorphisms. Sequencing reactions using universal infra red fluoresecent dye labelled M13 sequence oligonucleotides were then carried out on the PCR products. Sequencing products were resolved by polyacrylamide electrophoresis on LiCor 4200 L sequencers and the gel images were inspected manually.

### Statistical analysis

All marker data was first tested for the presence or absence of Hardy Weinberg equilibrium (HWE) in both case and control samples. If there was lack of HWE in the control sample the genotyping was repeated and the data from that marker was then rejected if HWE was still not observed. Single marker allelic associations were performed using standard chi-squared tests of 2 × 2 contingency tables. Linkage disequilibrium analysis was performed using Haploview [39]. The multi locus haplotype association analysis was performed using GENECOUNTING [40,41] and should any statistically significant asymptotic p-values occur, then an empirical p value would be calculated by permutation testing.

## Results

### Allelic, genotypic and haplotypic association studies

All of the markers were found be in Hardy Weinberg Equilibrium (p > 0.05). Reduplicated samples showed strong consistency in genotypes called.

**Figure 1:**
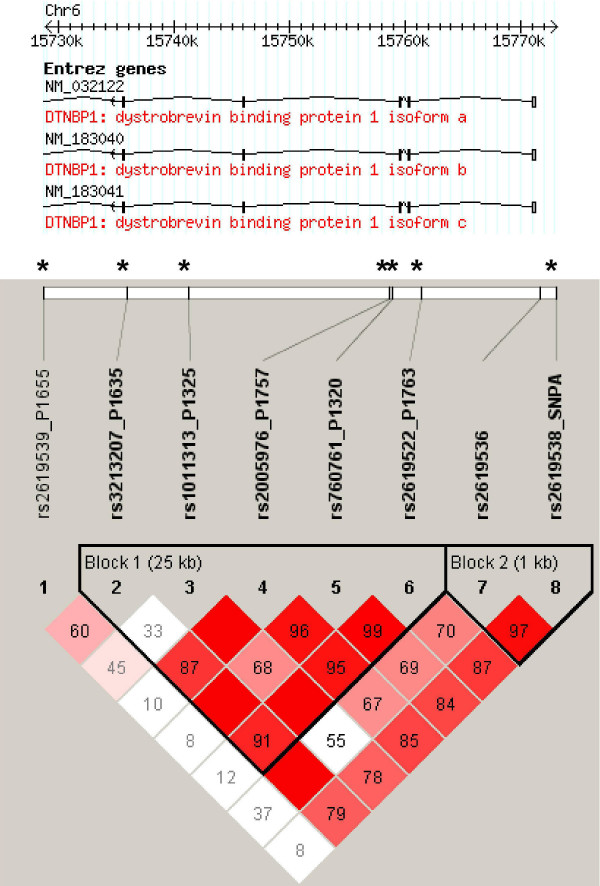
A Haploview generated diagram of the location of SNPs in the DNTBP1 gene that were genotyped in this study. SNPs marked with an asterisk have been found to be associated with schizophrenia in previous studies. Marker to marker D' statistics are shown below with LD blocks defined as solid spine of LD with a D' > 0.8.

Table 1 shows the allele frequencies and tests of association for the SNP markers. None of these tests produced a statistically significant association. Likewise, no genotypic associations were found. Pair-wise linkage disequilibrium was calculated between all pairs of markers using Haploview and the results are displayed in Figure 1. Tests of haplotypic association were carried out even though markers were individually negative and did not show a trend for significant association. A sliding window of two and three marker haplotypes did not reveal any significant associations. Resequencing of the randomly chosen individuals had 100% concordance rate with the results from Kbioscience which provided validity for the data.

**Table 1:** Genotype and allele frequencies and tests of association with schizophrenia on chromosome 6p22.3 at the *DTNBP1 *locus in cases and controls

Marker	Distance to next marker (bp)	Genotypes and frequencies			Allelic bases and frequencies		χ^2^	Significance (p =)
rs2619539 P1655	0	CC	GC	GG	C	G	0.0194	0.8891
Controls		68	135	64	271	263		
Schizophrenia		68	124	62	260	248		
rs3213207 P1635	7247	AA	AG	GG	A	G	0.248	0.6182
Controls		345	85	5	775	95		
Schizophrenia		305	71	3	682	78		
rs1011313 P1325	5330	CC	CT	TT	C	T	1.036	0.3088
Controls		356	70	6	782	82		
Schizophrenia		329	79	6	737	91		
rs2005976 P1757	17370	AA	GA	GG	A	G	0.949	0.3301
Controls		17	135	279	169	693		
Schizophrenia		8	119	254	135	627		
rs760761 P1320	330	CC	CT	TT	C	T	0.274	0.6006
Controls		274	135	17	683	169		
Schizophrenia		247	115	13	609	141		
rs2619522 P1763	2517	GG	TG	TT	G	T	0.427	0.5135
Controls		17	134	283	168	700		
Schizophrenia		11	128	275	150	678		
rs2619536	10198	CC	CT	TT	C	T	0.184	0.6678
Controls		5	80	344	90	768		
Schizophrenia		2	67	292	71	651		
rs2619538 SNPA	1362	TT	TA	AA	T	A	0.038	0.8462
Controls		134	208	88	476	384		
Schizophrenia		136	200	94	472	388		

## Discussion

The fact that we did not find any association between DTNBP1 SNPs and schizophrenia is consistent with our previous linkage studies which did not find evidence for linkage across the DTNBP1 region. However, linkage analysis is highly prone to the effects of heterogeneity of linkage and family size. Our own and other negative linkage studies can easily be explained as being due to an admixture of different subtypes of schizophrenia in different family samples. Tests of allelic association in case control samples should be more robust to the effects of locus heterogeneity and the power of a sample to detect allelic association becomes crucial. We calculated that our sample is large enough to detect a marker allele frequency difference of between 5% to 10% between cases and controls with a power of 0.99 at p < 0.05 and of 0.91 at p < 0.001 with an odds ratio of 2.00 assuming the minor marker allele frequency is less than 10%. With a high frequency bi-allelic SNP marker with an odds ratio of 2.00 the sample only had a 80% power to reach a p value of less than 0.05 under dominant, recessive or additive models if there was a 17% difference in allele frequencies (ie from 50% in controls to 67% in cases. It is possible therefore that if the DTNBP1 disease associated alleles are present in high frequency and only differ in frequency by, say about 5% between cases and controls then the proportion of DTNBP1 associated schizophrenic individuals in our sample may be too small to detect linkage disequilibrium.

When one considers the almost random way in which different studies report different alleles and haplotypes at DTBNBP1 to be associated with schizophrenia for example even within the Irish population. Then one could come to the conclusion that the positive results could all be random or false positive. Yet the original linkage evidence on chromosome 6 was very robust. The original positive allelic associations [11] were reported in a sample which had half or even less power than some of the other samples subsequently used which have been negative. At the same time apparent replications have been generated from small samples consisting of as few as 142 cases [16].

In the Straub study [11] the SNPs P1635 and P1320 were the two markers most strongly associated with schizophrenia whereas two of the three SNPs located in the interval between P1635 and P1320 were not associated. A German sample [14] also found evidence for association in two separate samples as well as in a combined sample with P1320 which gave a p value of p = 0.00068. We genotyped both of these strongly associated markers but still found no evidence of association.

The study by Williams and others on two samples from the UK and Ireland was negative for all the previously reported positive SNPs [17]. Sixteen novel SNPs were genotyped in these samples and none of these markers were significantly associated schizophrenia [17]. Haplotypic association analysis found a 3-marker haplotype composed of SNPs: SNP A, P1635, and P1655 that was associated with schizophrenia (p = 0.000056). All other significant haplotypes contained SNPA and either P1635 and/or P1655. Only one haplotype was increased in frequency in controls (p = 0.01) and this was found in both the Cardiff and Irish sub samples. The large difference in the strength of single marker versus haplotypic association in this study is unexpected and is inconsistent.

In our study we used genomic sequencing to check that the validity of some of the genotypes and haplotypes. No genetic variation in or around the DTNBP1 exons and its control regions have so far been found to have a convincing role in causing schizophrenia [42]. There are other genes and expressed sequences in the associated and linked region and these will also need resequencing in those individuals with at risk haplotypes. It is possible that resequencing DTNBP1 in a subgroup of subjects who have inherited the alleles responsible for any association might reveal a disease mutation. However, because different alleles and haplotypes have been found to be associated in the different studies, an explanation for these inconsistencies must first be found. This latter problem has now also been identified also by Mutsuddi and coauthors [13] who genotyped all the schizophrenia associated single-nucleotide polymorphisms reported in each of the studies in DNA samples from the CEPH-derived HapMap sample (CEU). Using this high-density reference map they found that that the haplotypes from each association study were inconsistent with regard to any single disease-associated haplotype at DTNBP1. Specifically, all five of the "replication" studies defined a positively associated haplotype that was different from that which was reported originally. They also showed that, in all six studies, the European-derived populations studied had haplotype patterns and frequencies that were consistent with the HapMap CEU samples [13].

If one considers the overall pattern of results it seems that the family based association studies obtain more significant results than the case control studies. One explanation for lack of replication by the case control studies is that the family based tests of association have not been able to distinguish very well between evidence of linkage and evidence of association. This is most likely to occur in family samples where positive linkage with schizophrenia has been found on chromosome 6 such as in the Irish and German samples. The reported evidence for "association" might therefore be a confirmation of linkage or mixture of both. Alternatively one could surmise that DTNBP1 associated schizophrenia occurs predominantly in familial cases of schizophrenia and that case control samples may therefore contain fewer cases of DTNBP1 associated schizophrenics. Our case control sample has failed to detect association at the CAPON and RGS4 loci and has implicated the EPSIN4 gene on 5q33, the UHMK1 gene on 1q22.3, the PCM1 gene on 8p22 and the FXYD6 gene on 11q22 with schizophrenia [40,43-47]. All these loci had all been implicated by prior linkage studies and confirmed by tests of association using case control samples without subdividing cases of schizophrenia by being family history positive or negative. Thus it is not clear that this distinction would make much difference to the outcome of genetic studies of DTNBP1. In genetic association studies as a whole it might be justified to discount certain family based studies as having failed to accurately fine map a disease gene by being unable to detect pure allelic association/linkage disequilibrium because of the presence of linkage in the family sample. In the case of the chromosome 6 schizophrenia linked region it now seems justifiable to consider that there could be genetic locus near by but still very close to DTNB1 that is causing susceptibility to schizophrenia. Alternatively an adequate explanation needs to be sought for the inconsistency in alleles and haplotypes found to be associated with schizophrenia.

## Competing interests

The author(s) declare that they have no competing interests.

## Authors' contributions

All authors read and approved the final manuscript.
